# Concentrations of Insulin-like Growth Factors and Insulin-like Growth Factor-Binding Proteins and Respective Gene Expressions in Children before and after Hematopoietic Stem Cell Transplantation

**DOI:** 10.3390/nu13124333

**Published:** 2021-11-30

**Authors:** Wojciech Strojny, Wojciech Czogała, Przemysław Tomasik, Mirosław Bik-Multanowski, Małgorzata Wójcik, Klaudia Miklusiak, Karol Miklusiak, Przemysław Hałubiec, Szymon Skoczeń

**Affiliations:** 1 Department of Pediatric Oncology and Hematology, University Children’s Hospital of Krakow, 30-663 Krakow, Poland; wojciech.strojny@mp.pl (W.S.); wojciech.czogala@uj.edu.pl (W.C.); 2 Department of Pediatric Oncology and Hematology, Faculty of Medicine, Jagiellonian University Medical College, 30-663 Krakow, Poland; 3 Department of Clinical Biochemistry, Faculty of Medicine, Jagiellonian University Medical College, 30-663 Krakow, Poland; p.tomasik@uj.edu.pl; 4 Department of Medical Genetics, Faculty of Medicine, Jagiellonian University Medical College, 30-663 Krakow, Poland; miroslaw.bik-multanowski@uj.edu.pl; 5 Department of Pediatric and Adolescent Endocrinology, Faculty of Medicine, Jagiellonian University Medical College, 30-663 Krakow, Poland; malgorzata.wojcik@uj.edu.pl; 6 Student Scientific Group of Pediatric Oncology and Hematology, Jagiellonian University Medical College, 30-663 Krakow, Poland; klaudia.mklk@gmail.com (K.M.); karolmiklusiak@gmail.com (K.M.); przemyslawhalubiec@gmail.com (P.H.)

**Keywords:** IGF, IGFBP, HSCT, children, microarray

## Abstract

Insulin-like growth factors (IGF-1 and IGF-2) and insulin-like growth factor-binding proteins (IGFBP-1 to -7) are involved in the regulation of cell proliferation and differentiation and may be associated with various metabolic parameters. The aim of our study was to compare levels of IGFs and IGFBPs and the expressions of their genes in children before and after hematopoietic stem cell transplantation (HSCT) to assess their potential as markers of late metabolic complications of HSCT. We also conducted additional comparisons with healthy controls and of correlations of IGF and IGFBP levels with anthropometric and biochemical parameters. We analyzed 19 children treated with HSCT and 21 healthy controls. We found no significant differences in the levels of IGFs and IGFBPs and expressions of their genes before and after HSCT, while IGF and IGFBP levels were significantly lower in children treated with HSCT compared with controls. We conclude that our results did not reveal significant differences between the levels of IGFs and IGFBPs before and after HSCT, which would make them obvious candidates for markers of late complications of the procedure in children. However, due to the very low number of patients this conclusion must be taken with caution and may be altered by further research.

## 1. Introduction

Hematopoietic stem cell transplantation (HSCT) is an important, life-saving treatment modality in children with cancer and selected non-neoplastic diseases [1,2,3]. Late complications of HSCT include various metabolic abnormalities, including obesity, dyslipidemia, and metabolic syndrome, which not only may affect the health status of long-term HSCT survivors but also can be important risk factors of cardiovascular disease in patients after HSCT [4,5,6]. Therefore, there is a need for biomarkers to target patients treated with HSCT who are at risk of developing late metabolic complications of the procedure, including obesity, so that their nutritional status can be monitored and appropriate interventions can be applied before their health is seriously compromised [7]. Identification of such markers is still a matter of research, and available data are very limited, particularly in pediatric populations.

Insulin-like growth factors (IGF-1 and IGF-2) and insulin-like growth factor-binding proteins (IGFBP-1 to -7) are involved in the regulation of cell proliferation and differentiation [8,9,10]. However, the mechanisms of these effects are still being investigated. The classic theory of IGF-1 being only a mediator of GH effects, and of IGFBPs being only the IGF transporters, was altered by accumulating data that revealed that IGFs have various insulin-like effects on peripheral tissues, including stimulation of cellular proliferation, differentiation, and inhibition of apoptosis, while IGFBPs may have opposite effects, and their balance may direct cells towards proliferation or apoptosis [11,12,13]. This may be related to altered levels of certain IGFs and/or IGFBPs in some cases of obesity or malnutrition [12]. The relationships between IGFs and IGFBPs and expressions of their genes and insulin resistance, type 2 diabetes, metabolic syndrome, hypertension, and obesity are subject to ongoing research, with new data continually emerging [14,15,16,17,18,19]. In our earlier research we investigated correlations between levels of IGFs and IGFBPs and obesity in children and expressions of genes related to lipid metabolism in children treated with HSCT [20,21].

The aim of our study was to compare levels of IGFs and IGFBPs in children treated with HSCT before and after the procedure, as well as the expressions of genes encoding these factors, in an attempt of preliminary assessment of these factors as possible biomarkers for late metabolic complications of HSCT in children. We also conducted additional analyses to compare the levels of IGFs and IGFBPs in children treated with HSCT and a group of healthy controls and to assess correlations between the levels of IGFs and IGFBPs and a number of anthropometric and biochemical parameters.

## 2. Materials and Methods

### 2.1. Study Population

The study included two groups: children treated with HSCT and healthy controls. The HSCT group included 19 patients (mean age 11 years; 2.15 ± 19.2; 15 boys and 4 girls) consecutively referred for treatment to the Stem Cell Transplantation Centre of the University Children’s Hospital in Krakow. Indications for HSCT were neoplastic diseases (84%; median time from diagnosis 2.6 years; all except for one patient were in complete remission) or non-neoplastic diseases (16%; median time from diagnosis 3.2 years). For details of indications, see Table S1. All HSCT procedures were allogeneic (1 from matched family donor, 6 from matched sibling donor, and 12 from matched unrelated donor) and myleoablative conditioning was used (busulfan-based in 11 patients, treosulfan-based in 2 patients, and etoposide plus total body irradiation in 6 patients). In 7 children (37%) in the post-HSCT group graft-versus-host disease (GvHD) was diagnosed, and 14 (74%) children in this group were treated with systemic corticosteroids for the treatment of HSCT complications.

The control group consisted of 21 healthy children (mean age 12 years; 6.8 ± 16.8; 12 girls and 9 boys) recruited from family donors, siblings of patients treated with HSCT, and children of medical staff. All controls had unremarkable medical history and no signs or symptoms of acute or chronic diseases.

### 2.2. Study Protocol

The assessments in the study group were performed before the transplantation (pre-HSCT group) and after the procedure (post-HSCT group; median time after the procedure 6.4 months; range 6–21.4 months). The control group was assessed once—at enrollment to the study.

Blood for measurements was taken in the morning (fasting) to test tubes containing aprotinin. We measured blood concentrations of glucose, insulin, adiponectin, apelin, cholecystokinin, fibroblast growth factor 21, glucagon-like peptide-1, ghrelin, leptin, leptin receptor, resistin, and visfatin: The measurements were performed before (fasting) and at 60 and 120 min of the standard oral glucose tolerance test (OGTT) with 1.75 g of anhydrous glucose/kg body weight (max. 75 g). Blood samples were collected once—after the patient was enrolled to the study—to aprotinin-containing tubes. The tubes were delivered to the laboratory immediately and were centrifuged for 15 min at 3000 rpm using a horizontal rotor. Plasma samples for measurements of insulin, total IGF-1 and IGF-2, and IGFBP-1, -2, -3, -4, -6, and -7 were stored at −80 °C until the time of assay.

Height, weight, and waist circumference (WC) were measured by an anthropometrist. Body mass index (BMI) and BMI percentile/SDS were calculated using online WHO BMI calculators [22]. Their values were compared with local reference standards (WC) or WHO reference values (BMI and percentile/SDS).

### 2.3. Assays

Total levels of IGFs and IFGBPs were measured using ELISA kits. The following kits were used: IGF-1, Labor Diagnostika (Nord GmbH & Co.KG, Bargteheide, Germany; dynamic detection range was 9.75 ng/mL–600 ng/mL, sensitivity: 9.75 ng/mL, intraassay variance up to 7.4%, intraassay variance up to 14.8%). IGF-2 Mediagnost (Reultingen, Germany; assay detection range 0.06–3609 ng/mL, sensitivity 0.02 ng/mL, intra- and interassay variance < 10%); IGFBP-1, IGFBP-2, IGFBP-3, and IGFBP-6, Mediagnost (Reultingen, Germany; for IGF BP-2 assay detection range 0.2–1680 ng/mL, sensitivity 0.2 ng/mL, intra- and interassay variance < 0%; for IGFBP-3 assay detection range 0.03–15150 ng/mL, sensitivity 0.03 ng/mL, intra and interassay variance < 10%; for IGFBP-6 assay detection range 0.026–500 ng/mL, sensitivity 0.026 ng/mL, intra- and interassay variance < 10%); IGFBP-4 and IGFBP-7, FineTest (WuHan, China; for IFGBP-4 assay detection range 3.125–200 ng/mL, sensitivity 1.875 ng/mL, intraassay CV < 8%, interassay CV < 10%; for IGFBP-7 assay detection range 31.25–2000 pg/mL, sensitivity 18.75 pg/mL, intraassay CV < 8%, interassay CV < 10%).

The following assays were used for biochemical measurements: glucose—Vitros 5.1 dry chemistry analyzer (Johnson & Johnson, United Kingdom; Department of Clinical Biochemistry, Polish-American Institute of Pediatrics, Poland); insulin—radioimmunometry (sensitivity: 1 µU/mL, inter-series precision: CV < 6.5%, intra-series precision: CV < 2.1%) (BioSource Company Europe S.A, Nivelles, Belgium); leptin—enzyme-amplified sensitivity immunoassay (sensitivity: 0.1 ng/mL, inter-series precision: CV < 9.0%, intra-series precision: CV < 3.6%) (BioSource; Nivelles, Belgium); soluble leptin receptor—enzyme immunoassay (sensitivity: 0.04 ng/mL, inter-series precision: CV < 9.8%, intra-series precision: CV < 7.2%) (BioVendor Research and Diagnostic Products, Brno, Czech Republic); adiponectin, apelin, cholecystokinin, glucose-like peptide 1, ghrelin, and resistin—commercial assays (Phoenix Pharmaceuticals. Inc., Burlingame, CA, USA); fibroblast growth factor-21—commercial assay (Millipore corporation, Burlington, MA, USA); visfatin—enzyme immunoassay (Phoenix Pharmaceuticals, Inc., Burlingame, CA, USA).

### 2.4. Microarray Analysis

Whole genome expression in peripheral blood leukocytes was assessed using GeneChip Human Gene 1.0 ST Array (Affymetrix, Santa Clara, CA, USA), according to the manufacturer’s protocol. Total RNA extraction was performed using RiboPure Blood Kit (Ambion, Life Technologies, Carlsbad, CA, USA). Whole transcript microarray experiment was performed according to the manufacturer’s protocol (GeneChip Whole Transcript sense Target Labeling Assay Manual, Version 4). DTT data were transferred by Transfer Tool software (Affymetrix). Chip quality was assessed according to the Affymetrix guidelines. Raw data were processed using model-based expression index implemented in dChip. After background subtraction, the data were normalized using quintile normalization. The signal was taken as the measure of mRNA abundance derived from the level of gene expression. The GeneCards Human Genes Database [23] was used to analyze the function of genes.

### 2.5. Statistical Analysis

Continuous clinical and biochemical data are presented as mean ± SD or median as appropriate. Categorical data are presented as frequencies (*N*) and proportions (%). The Shapiro–Wilk test was used to estimate the normality of continuous data. Student’s t-test (for normally distributed variables) or Mann–Whitney test (for non-normally distributed variables) were used to assess differences between independent groups. Student’s t-test for paired samples (for normally distributed variables) or Wilcoxon’s rank sum test (for non-normally distributed variables) were used to assess differences between paired groups. Spearman’s rank correlation coefficient (r) was used to assess correlations between continuous variables. Two-sided *p* values < 0.05 were considered statistically significant. The statistical analyses were performed using Statistica 13 software (StatSoft).

The microarray data were preprocessed using the R/Bioconductor package (Bioconductor, USA). Robust multiarray average (RMA) was used for normalization. Principal component analysis (PCA), relative log expression (RLE), and normalized unscaled standard error (NUSE) plots were used for quality control. Moderated t-tests were used to detect probes with different expressions in different groups; R/Bioconductor limma package was used. The assumption was made that log2 transformed gene expression levels have a normal distribution and that variation between the groups is of comparable magnitude. To control for the false discovery rate (FDR), multiple testing correction (Benjamini–Hochberg procedure) was applied. Significantly different expression in the probe sets was defined as multiple comparison-corrected two-sided *p* value < 0.05.

### 2.6. Ethical Issues

The study protocol was approved by the permanent Ethical Committee for Clinical Studies of the Medical College of Jagiellonian University, Kraków, Poland. All parents, adolescent patients, and adult patients provided informed consent in writing before enrollment to the study. The sponsoring institutions had no influence on study design, sample and data collection, data analysis and interpretation, process of writing of the manuscript, or the decision to submit the manuscript. Protection measures of rights of minors and persons with intellectual disabilities were implemented. The study was planned and conducted in accordance with The Code of Ethics of the World Medical Association (Declaration of Helsinki).

## 3. Results

The anthropometric parameters of the pre-HSCT and post-HSCT groups are presented in Table 1, and respective biochemical parameters are presented in Table 2.

### 3.1. IGF-1 and IGF-2

In a pooled analysis of all children treated with HSCT, we found significant correlations between IGF-1 levels and several anthropometric parameters (height, weight, waist circumference cm/percentile, age, BF kg/%, LBM, TBW) (Table 3). Some differences were found in a sub-analysis of correlations with the pre-HSCT and post-HSCT groups (Tables S2 and S3). We found no significant correlations between IGF-2 levels and any of the above anthropometric parameters both in a pooled analysis and in the pre-HSCT and post-HST groups (Tables S2 and S3). We found significant differences of several biochemical parameters (apelin, cholecystokinin, glucagon-like peptide 1, leptin, and visfatin) between the pre-HSCT and post-HSCT groups (Table 2), but we found no significant correlations between IGF-1 or IGF-2 or between any of the biochemical parameters in a pooled analysis (Table S4), and we found only a few significant correlations in the pre-HSCT and post-HSCT groups (Tables S5 and S6).

**Figure 1:**
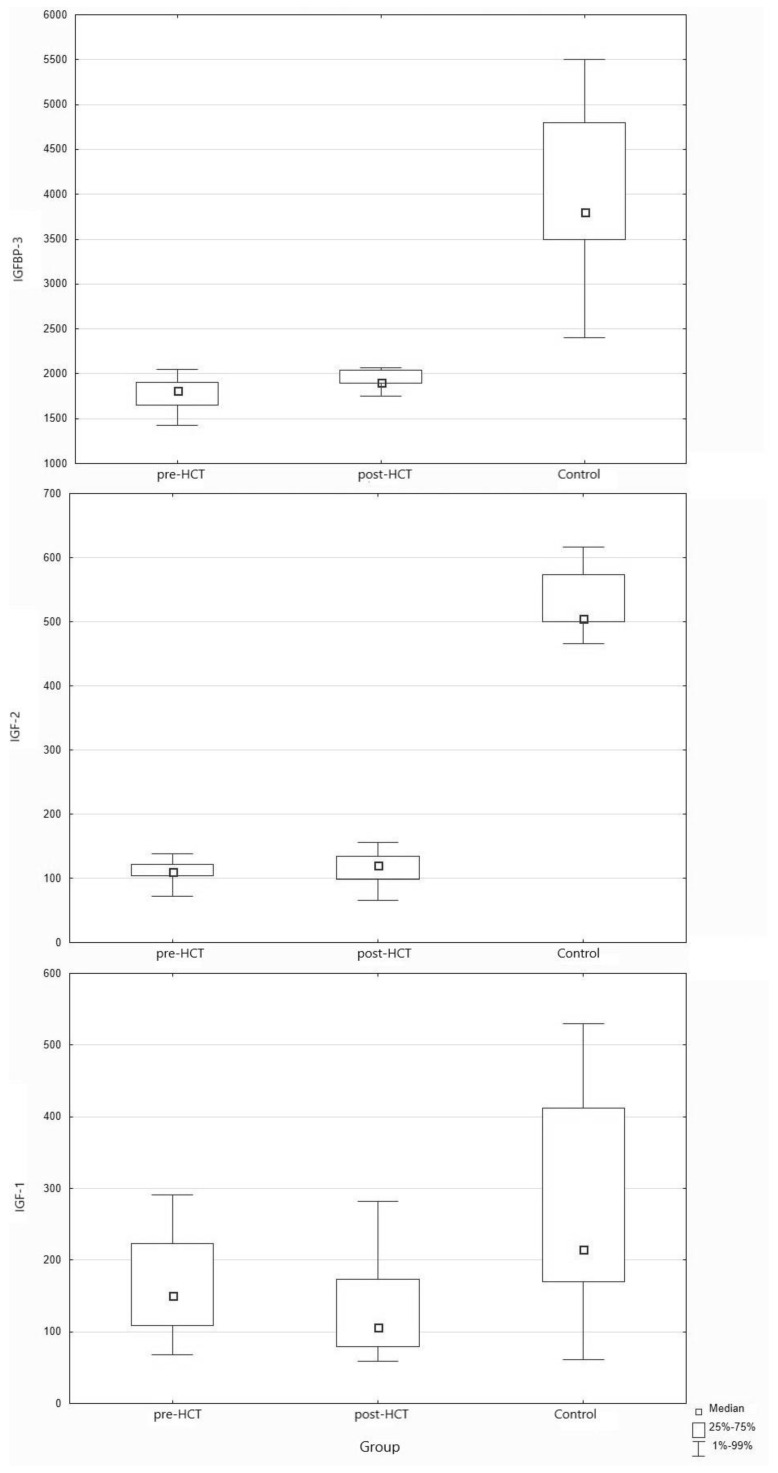
Concentrations of IGF-1, IGF-2 and IGFBP-3 (ng/mL) in pre-HSCT group, post-HSCT group, and healthy controls. HSCT, hematopoietic stem cell transplantation; IGF, insulin growth factor; IGFBP, insulin growth factor-binding protein.

We found no significant differences in the levels of IGF-1 or IGF-2 before and after the transplantation procedure in the HSCT group (Table 4). The levels of IGF-1 and IGF-2 were significantly lower in the pre-HSCT and post-HSCT groups compared with the control group (Table 5 and Table 6, Figure 1). We also found that IGF-1 levels in most patients (84.21% in the pre-HSCT group and 78.95% in the post-HSCT group) were within the well-established reference values published elsewhere (with a few exceptions that were minimally above or below the limit) [24,25,26], while IGF-2 levels in most patients (78.95% in the pre-HSCT group and 73.68% in the post-HSCT group) were below the published reference values both before and after HSCT.

### 3.2. IGF-Binding Proteins

We found several significant correlations between IGFBP-1 levels and several anthropometric parameters in a pooled analysis and in the pre-HSCT and post-HSCT groups (Tables S7–S9).

We found no significant correlations between IGFBP-2, IGFBP-3, IGFBP-4, IGFBP-5, IGFBP-6, or IGFBP-7 levels and any of the anthropometric or biochemical parameters in a pooled analysis, although numerous significant correlations were found in the pre-HSCT and post-HSCT groups (Tables S10–S12). Moreover, IGFBP-3 levels in most patients (89.47% in the pre-HSCT group and 78.95% in the post-HSCT group) were below the reference values published elsewhere [25,26].

### 3.3. IGF and IGFBP Gene Expressions

**Figure 2:**
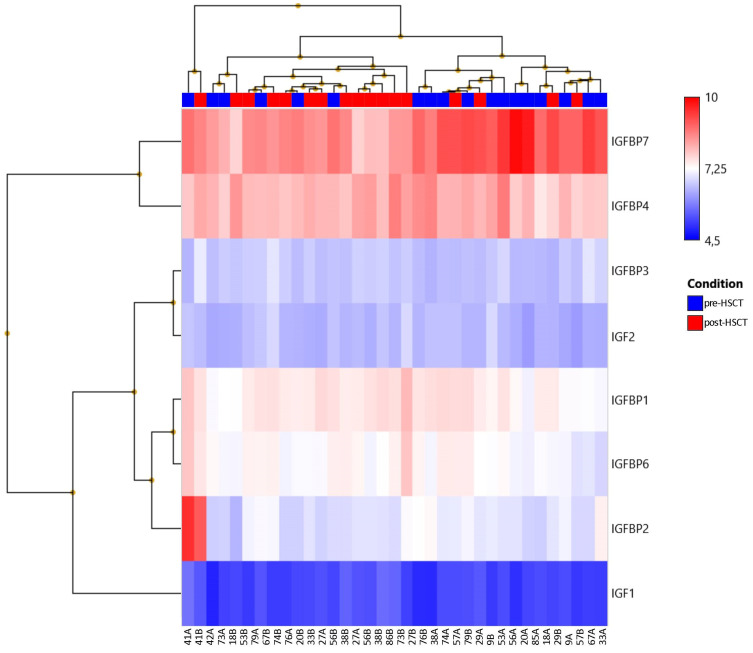
Expressions of genes encoding IGFs and IGFBPs in the pre-HSCT group and post-HSCT group. HSCT, hematopoietic stem cell transplantation; IGF, insulin growth factor; IGFBP, insulin growth factor-binding protein.

We found no significant differences in the expressions of IGF or IGFBP genes between the pre-HSCT and post-HSCT groups (Table 7, Figure 2).

## 4. Discussion

In our study, we found no significant differences in the levels of IGFs and IGFBPs before and after HSCT in children, and no significant differences in expressions of their genes before and after the procedure were found. To our knowledge, this is the first such report in the literature.

We found significant correlations between IGF-1 and IGFBP-1 levels and several anthropometric parameters. We found significant positive correlations between IGF-1 levels and height, weight, waist circumference, age, BF, LBM, and TBW, while no correlations were found with ECW and BMI. The levels of IGFBP-1 showed significant negative correlations with height, weight, waist circumference, age, BMI, and BF. No significant correlations with any of the anthropometric parameters were found for the levels of IGF-2 and IGFBP 2–7. We also found no significant differences in the levels of IGF-1 or IGF-2 before and after HSCT, and the levels of IGF-1 and IGF-2 were significantly lower in the pre-HSCT and post-HSCT group compared with the controls. We also found significant differences of apelin, cholecystokinin, glucagon-like peptide 1, leptin, and visfatin between the pre-HSCT and post-HSCT groups. We found numerous significant correlations between several anthropometric and biochemical parameters and levels of individual IGFs and IGFBPs in the pre-HSCT and post-HSCT groups, which were not present in a pooled analysis of the group treated with HSCT. These findings are in line with prior reports on metabolic complications of HSCT in children [27], but their importance cannot be formally assessed in our study due to the very small sample and short follow up. Nevertheless, nutritional status of HSCT recipients is an important issue in the management of these patients with respect to metabolic abnormalities affecting the results of treatment and its complications. The importance of appropriate nutritional support, including enteral and parenteral nutrition, has been appreciated in systematic reviews and meta-analyses [28,29].

When interpreting the IGF levels in patients treated with cancer, it must be noted that their effects are not restricted to metabolism. IGFs have also been implicated in carcinogenesis, and their altered levels were found in various types of cancer, including patients treated with HSCT, and the role of signaling pathways in the development of cancer is a matter of ongoing research [30,31,32,33]. The low levels of IGF-1 before HSCT are consistent with previous findings in children with acute leukemia or non-neoplastic hematological diseases [34,35,36,37]. IGF-1 levels were also investigated as possible predictors of sinusoidal obstruction syndrome (SOS) in patients treated with HSCT [38]. Therefore, it should be assumed that their levels are very likely affected by both the underlying condition and the treatment itself. However, we found low absolute levels of IGFs in patients treated with HSCT and no change in the levels between the pre-HSCT and post-HSCT status. This might be because the patients were qualified for transplantation while in complete remission, and the remission was also maintained at the follow-up, but definitive conclusions cannot be drawn due to limited follow-up.

The National Institutes of Health (NIH) define a prognostic biomarker as a biomarker used to identify the likelihood of a clinical event and to have a favorable or unfavorable effect of an exposure, and the methodology for defining biomarkers is constantly being developed [39,40]. The use of IGF-2 as biomarkers of obesity or therapeutic targets was the subject of preliminary research [15,19,41]. The possible use of IGF-1 as a biomarker of metabolic complications of HSCT has been recently included in a review by Morrello et al. [42], but the data were preliminary and inconclusive. To our knowledge, no such data are available for children treated with HSCT.

The usefulness of IGFs and IGFPBs as biomarkers of possible future metabolic abnormalities should be based on the relative changes in their levels before and after HSCT rather than their absolute levels compared with controls or reference values. The possible use of any of the growth factors as a biomarker would require any consistent change (increase or decrease) between two time-points—before and after the procedure. However, no such change was found in our patients for any of the IGFs or IGFBPs. We found some changes in the levels of these factors, but none of them reached statistical significance, and there was no consistent pattern to the changes (IGF levels increased slightly, while IGFBP levels revealed a whole range of changes from slight increase in IGFBP 1, 3, and 6, through major increases in IGFBP 2 and 5, to a decrease in IGFBP 7).

Preliminary research indicated a substantial genetic contribution to variations in IGF and IGFBP levels in adults [43], and attempts were made to identify genetic variants associated with IGF-1 levels and their possible predictive value in various types of cancer [44,45]. In a retrospective study, Ebessen et al. [37] investigated single nucleotide polymorphisms (SNPs) of the genes encoding IGF-1 and IGFBP-3 as possible predictors of toxicities and survival after HSCT. To our knowledge, this was the first such study in the literature, and they found the results promising and worth further research. We assessed the expressions of the genes encoding IGFs and IGFBPs before and after HSCT, and we found no significant differences in gene expressions and no distinguishable pattern of trend of changes.

Our study has several limitations, including single-center design, small patient groups, and short follow-up. Therefore, our findings are preliminary and might be altered by later research on larger patient groups.

## 5. Conclusions

In conclusion, we found that levels of IGFs and IGFBPs showed no significant differences before and after HSCT in children, and no significant differences in expressions of their genes before and after the procedure were found. However, the observed correlations of the levels of IGFs and IGFBPs with anthropometric and biochemical parameters were consistent with their possible roles in metabolic regulation. Based on our results, it could not be concluded that IGFs and IGFBPs or their gene expressions may be used as markers of late complications of HSCT in children because of inconsistent and a statistically non-significant pattern of their differences before and after the procedure. However, in view of the very small size and short duration of our study, this must be taken with much caution, as larger studies with longer follow-up may lead to different conclusions.

## Figures and Tables

**Table 1:** Anthropometric characteristics of the study group (mean ± standard deviation).

Baseline Characteristic	Pre-HSCT (N = 19)	Post-HSCT (N = 19)	*p* Value
BMI percentile	59.91 ± 31.01	46.57 ± 34.25	0.12
Blood pressure systolic/diastolic	109 ± 9.89/69.11 ± 11.74	104.84 ± 10.25/63.05 ± 11.97	0.34/0.09
Height (mm)	1346.21 ± 294.77	1390.37 ± 260.81	0.02
Weight (kg)	36.32 ± 17.88	36.04 ± 15.14	0.59
Waist circumference (cm/percentile)	66.1 ± 12.08/56.47 ± 28.95	64.66 ± 10.48/48.33 ± 26.03	0.26/0.33
Age (months)	115.26 ± 63.11	126.42 ± 59.95	0.002
BF (kg/%)	6.08 ± 6.44/14.34 ± 10.53	6.48 ± 5.44/16.21 ± 8.88	0.94/0.88
ECW	9.16 ± 3.12	9.3 ± 4.79	0.7
LBM	28.8 ± 12.46	28.6 ± 10.74	0.97
TBW	21.2 ± 9.03	21 ± 7.79	0.86

BF, body fat; BMI, body mass index; ECW, extracellular body water; LBW, lean body mass; TBW, total body water.

**Table 2:** Biochemical parameters of the study group (mean ± standard deviation).

Baseline Characteristic	Pre-HSCT (N = 19)	Post-HSCT (N = 19)	*p* Value
Glucose T0 (mmol/L)	4.5 ± 0.56	4.54 ± 0.52	0.84
Glucose T60 (mmol/L)	6.04 ± 1.08	5.75 ± 0.8	0.43
Glucose T120 (mmol/L)	5.04 ± 1.14	5.51 ± 0.1	0.16
Insulin T0 (μIU/L)	13.2 ± 9.36	8.16 ± 4.22	0.2
Insulin T60 (μIU/L)	43.6 ± 25.39	29.43 ± 28.04	0.08
Insulin T120 (μIU/L)	26.07 ± 22.76	19.45 ± 14.95	0.26
Adiponectin T0 (μg/mL)	4.29 ± 3.87	3.83 ± 2.44	0.69
Adiponectin T60 (μg/mL)	4.30 ± 3.72	2.56 ± 1.49	0.09
Adiponectin T120 (μg/mL)	3.65 ± 2.61	2.25 ± 0.96	0.22
Apelin T0 (pg/mL)	1.05 ± 0.49	8.89 ± 11.3	0.008
Apelin T60 (pg/mL)	1.38 ± 1.41	4.74 ± 5.14	0.008
Apelin T120 (pg/mL)	1.16 ± 0.47	5.21 ± 5.98	0.009
Cholecystokinin T0 (nmol/L)	1.15 ± 0.63	4.74 ± 6.73	0.004
Cholecystokinin T60 (nmol/L)	1.01 ± 0.44	4.68 ± 5.93	0.001
Cholecystokinin T120 (nmol/L)	1.16 ± 0.59	4.12 ± 4.68	0.0009
Fibroblast growth factor 21 T0 (ng/mL)	223.28 ± 200.27	139.12 ± 164.17	0.03
Fibroblast growth factor 21 T60 (ng/mL)	222.89 ± 184.25	132.04 ± 156	0.04
Fibroblast growth factor 21 T120 (ng/mL)	280.83 ± 227.81	166.5 ± 171.78	0.01
Ghrelin T0 (pg/mL)	510.49 ± 98.69	679.52 ± 375.3	0.04
Ghrelin T60 (pg/mL)	483.91 ± 105.59	660.73 ± 276.52	0.02
Ghrelin T120 (pg/mL)	514.42 ± 101.55	676.9 ± 324.46	0.06
Glucagon-like peptide-1 T0 (nmol/dL)	0.68 ± 0.51	1.57 ± 1.39	0.003
Glucagon-like peptide-1 T60 (nmol/dL)	0.57 ± 0.21	1.23 ± 0.78	0.007
Glucagon-like peptide-1 T120 (nmol/dL)	0.66 ± 0.48	1.19 ± 0.94	0.01
Leptin T0 (ng/dL)	11.56 ± 19.54	13.42 ± 24.61	0.4
Leptin T60 (ng/dL)	12.85 ± 23.01	7.41 ± 18.48	0.009
Leptin T120 (ng/dL)	13.11 ± 23.63	8.34 ± 22.52	0.006
Leptin receptor T0 (μg/L)	22.02 ± 17.41	28.21 ± 22.41	0.31
Leptin receptor T60 (μg/L)	22.38 ± 16.30	30.93 ± 20.80	0.03
Leptin receptor T120 (μg/L)	21.47 ± 25.81	30.17 ± 20.22	0.03
Resistin T0 (ng/mL)	3.8 ± 3	3.43 ± 1.99	0.77
Resistin T60 (ng/mL)	3.29 ± 1.89	3.66 ± 1.7	0.8
Resistin T120 (ng/mL)	3.44 ± 1.7	3.58 ± 1.61	0.95
Visfatin T0 (ng/mL)	6.07 ± 2.93	16.29 ± 14.76	0.004
Visfatin T60 (ng/mL)	7.23 ± 4.69	14.21 ± 12.46	0.07
Visfatin T120 (ng/mL)	6.95 ± 2.23	13.66 ± 12.1	0.06

T0, measured at fasting; T60, measured at 60 min; and T120, measured at 120 min of the standard oral glucose tolerance test (OGTT).

**Table 3:** Correlations of anthropometric parameters with IGF-1 and IGF-2 concentrations.

	IGF-1		IGF-2	
Anthropometric Parameters	Spearman Correlation Coefficient r	*p* Value	Spearman Correlation Coefficient r	*p* Value
BMI percentile	0.034	0.989	0.08	0.940
Blood pressure systolic/diastolic	0.227/0.134	0.439	−0.078/0.077	0.940
Height mm	0.533	0.024	0.148	0.940
Weight kg	0.556	0.024	0.08	0.940
Waist circumference cm/percentile	0.496/0.076	0.039	0.096/−0.007	0.536
BMI WHO	0.368	0.107	−0.05	0.940
BF kg/%	0.55/0.49	0.047	0.184/0.228	0.940
ECW	0.497	0.084	0.104	0.940
LBM	0.563	0.039	0.106	0.940
TBW	0.564	0.039	0.101	0.940

BF, body fat; BMI, body mass index; ECW, extracellular body water; LBW, lean body mass; TBW, total body water.

**Table 4:** Concentrations of IGFs and IGFBPs in pre-HCT and post-HSCT groups.

Parameters	Pre-HSCT (ng/mL)	Post-HSCT (ng/mL)	*p* Value
IGF-1	97.85	119.2	0.088
IGF-2	120.16	137	0.407
IGFBP-1	50.74	59.8	0.08
IGFBP-2	198.1	424.3	0.163
IGFBP-3	1741.52	1943.34	0.08
IGFBP-4	40.39	50.88	0.936
IGFBP-6	56.31	98.39	0.08
IGFBP-7	32.44	27.18	0.481

**Table 5:** Concentrations of IGFs and IGFBPs in pre-HSCT group and healthy controls.

Parameters	Pre-HSCT (ng/mL)	Healthy Controls (ng/mL)	*p* Value
IGF-1	97.85	253.2 ± 145.6	0.032
IGF-2	120.16	828.68 ± 908.7	<0.001
IGFBP-1	50.74	8.03 ± 9.13	0.704
IGFBP-2	198.1	139.89 ± 122.58	0.303
IGFBP-3	1741.52	3965.8 ± 846.4	<0.0001
IGFBP-4	40.39	26.76 ± 18.74	0.131
IGFBP-6	56.31	148.79 ± 65.09	0.0003
IGFBP-7	32.44	67.86 ± 47.24	0.363

**Table 6:** Concentrations of IGFs and IGFBPs in healthy controls and post-HSCT group.

Parameters	Healthy Controls (ng/mL)	Post-HSCT (ng/mL)	*p* Value
IGF-1	270.13 ± 140	119.2	0.008
IGF-2	830.74 ± 908.2	137	<0.001
IGFBP-1	8.03 ± 9.13	59.8	0.134
IGFBP-2	139.89 ± 122.58	424.3	0.026
IGFBP-3	4821.05 ± 3719	1943.34	0.757
IGFBP-4	26.76 ± 18.74	50.88	0.373
IGFBP-6	148.79 ± 65.09	98.39	0.124
IGFBP-7	67.86 ± 47.24	27.18	0.285

**Table 7:** Gene expression differences between the pre-HSCT and post-HSCT group (log2 mean ± standard deviation), representing the recorded signal intensity of the probes.

Parameters	Pre-HSCT	Post-HSCT	*p* Value
IGF-1	1.74 ± 0.49	8.4 ± 0.56	0.39
IGF-2	96.8 ± 10.7	96.7 ± 11.35	0.81
IGFBP-1	7.53 ± 0.27	7.59 ± 0.23	0.76
IGFBP-2	7.02 ± 0.54	6.96 ± 0.44	0.81
IGFBP-3	6.58 ± 0.14	6.69 ± 0.15	0.21
IGFBP-4	8 ± 0.3	8.13 ± 0.22	0.16
IGFBP-5	5.18 ± 0.17	5.21 ± 0.15	0.84
IGFBP-6	7.31 ± 0.22	5.21 ± 0.24	0.85
IGFBP-7	1.74 ± 0.49	8.4 ± 0.56	0.39

## Data Availability

The datasets generated for this study are available on request from the corresponding author.

## Supplementary Materials

The following are available online at https://www.mdpi.com/article/10.3390/nu13124333/s1, Table S1: Indications for HSCT, Table S2: Correlations of anthropometric parameters with IGF-1 and IGF-2 concentrations in the pre-HSCT group, Table S3: Correlations of anthropometric parameters with IGF-1 and IGF-2 concentrations in the post-HSCT group, Table S4: Correlations of biochemical parameters with IGF-1 and IGF-2 concentrations (pooled analysis), Table S5: Correlations of biochemical parameters with IGF-1 and IGF-2 concentrations in the pre-HSCT group, Table S6: Correlations of biochemical parameters with IGF-1 and IGF-2 concentrations in the post-HSCT group, Table S7: Correlations of anthropometric parameters with IGFBP concentrations (pooled analysis), Table S8: Correlations of anthropometric parameters with IGFBP concentrations in the pre-HSCT group, Table S9: Correlations of anthropometric parameters with IGFBP concentrations in the post-HSCT group, Table S10: Correlations of biochemical parameters with IGFBP concentrations (pooled analysis), Table S11: Correlations of biochemical parameters with IGFBP concentrations in the pre-HSCT group, Table S12: Correlations of biochemical parameters with IGFBP concentrations in the post-HSCT group.
