# Supplementary material for: Concentrations of Insulin-like Growth Factors and Insulin-like Growth Factor-Binding Proteins and Respective Gene Expressions in Children before and after Hematopoietic Stem Cell Transplantation
Source: Nutrients. 2021 Nov 30;13(12):4333. doi: 10.3390/nu13124333 (PMC8709367; doi:10.3390/nu13124333)
Supplement: Supplementary file 1 [file nutrients-13-04333-s001.zip › nutrients-1405437-supplementary.pdf]

Table S1. Indications for HSCT.

Diagnosis	Number (total *N* = 19)
**Neoplastic diseases**	**16**
Acute lymphoblastic leukemia	10
Acute myeloblastic leukemia	5
Mielodysplastic syndrome	1
**Non-neoplastic diseases**	**3**
Chronic granulomatous disease	1
Autoimmune lymphoproliferative syndrome	1
Hyper IgM syndrome	1

Table S2. Correlations of anthropometric parameters with IGF-1 and IGF-2 concentrations in the pre-HSCT group.

	IGF-1		IGF-2	
**Anthropometric parameters**	**Spearman correlation coefficient r**	**P value**	**Spearman correlation coefficient r**	**P value**
BMI percentile	0.231	0.389	0.361	0.128
Blood pressure systolic/diastolic	0.049/-0.246	0.858/0.356	0.157/0.174	0.520/0.477
Height mm	0.673	0.004	0.188	0.442
Weight kg	0.661	0.005	0.237	0.328
Waist circumference cm/percentile	0.744/0.298	0.0009/0.347	0.293/0.422	0.223/0.118
Age months	0.649	0.007	0.182	0.455
BMI WHO	0.505	0.046	0.312	0.191
BF kg/%	0.541/0.587	0.085/0.058	0.188/0.122	0.538/0.692
ECW	0.618	0.043	0.157	0.608
LBM	0.491	0.125	0.300	0.319
TBW	0.491	0.125	0.300	0.319

Table S3. Correlations of anthropometric parameters with IGF-1 and IGF-2 concentrations in the post-HSCT group.

	IGF-1		IGF-2	
**Anthropometric parameters**	**Spearman correlation coefficient r**	**P value**	**Spearman correlation coefficient r**	**P value**
BMI percentile	-0.214	0.39	0.361	0.128
Blood pressure systolic/diastolic	0.325/0.3	0.19/0.3	-0.043/0.04	0.861/0.866
Height mm	0.578	0.01	0.036	0.885
Weight kg	0.518	0.028	-0.05	0.829
Waist circumference cm/percentile	0.36/-0.18	0.138/0.53	-0.097/-0.228	0.693/0.414
Age months	0.457	0.057	-0.074	0.764
BMI WHO	0.121	0.633	-0.28	0.245
BF kg/%	0.67/0.536	0.017/0.07	0.222/0.281	0.467/0.352
ECW	0.525	0.08	0.072	0.816
LBM	0.65	0.02	0.114	0.7
TBW	0.652	0.022	0.11	0.7

Table S4. Correlations of biochemical parameters with IGF-1 and IGF-2 concentrations (pooled analysis).

	IGF-1		IGF-2	
**Biochemical parameters**	**Spearman correlation coefficient r**	**P value**	**Spearman correlation coefficient r**	**P value**
Glucose T0	0.059	0.989	0.062	0.940
Glucose T60	0.297	0.307	0.029	0.940
Glucose T120	0.323	0.270	0.377	0.505
Insulin T0	0.468	0.084	0.158	0.940
Insulin T60	0.418	0.102	-0.095	0.940
Insulin T120	0.421	0.102	0.047	0.940
Adiponectin T0	-0.007	0.989	-0.016	0.946
Adiponectin T60	0.126	0.823	0.05	0.940
Adiponectin T120	0.206	0.588	-0.047	0.940
Apelin T0	-0.026	0.989	0.339	0.505
Apelin T60	0.03	0.989	0.14	0.940
Apelin T120	0.003	0.989	0.165	0.940
Cholecystokinin T0	0.085	0.934	0.239	0.875
Cholecystokinin T60	0.09	0.934	0.182	0.940
Cholecystokinin T120	0.13	0.823	0.27	0.875
Fibroblast growth factor 21 T0	0.236	0.464	0.11	0.940
Fibroblast growth factor 21 T60	0.275	0.408	0.064	0.940
Fibroblast growth factor 21 T120	0.226	0.527	0.03	0.940
Ghrelin T0	-0.231	0.439	-0.02	0.940
Ghrelin T60	-0.183	0.600	0.066	0.940
Ghrelin T120	-0.193	0.588	0.079	0.940
Glucagon-like peptide-1 T0	-0.002	0.989	0.17	0.940
Glucagon-like peptide-1 T60	0.054	0.989	0.144	0.940
Glucagon-like peptide-1 T120	0.096	0.934	0.202	0.940
Leptin T0	-0.04	0.989	-0.244	0.875
Leptin T60	-0.015	0.989	-0.39	0.505
Leptin T120	-0.046	0.989	-0.424	0.505
Leptin receptor T0	-0.342	0.150	-0.117	0.940
Leptin receptor T60	-0.398	0.105	-0.164	0.940
Leptin receptor T120	-0.412	0.102	-0.15	0.940
Resistin T0	-0.014	0.989	0.13	0.940
Resistin T60	0.085	0.934	-0.008	0.966
Resistin T120	0.18	0.627	0.029	0.940
Visfatin T0	0.047	0.989	0.062	0.940
Visfatin T60	0.29	0.366	0.143	0.940
Visfatin T120	0.07	0.989	-0.026	0.940

Table S5. Correlations of biochemical parameters with IGF-1 and IGF-2 concentrations in the pre-HSCT group.

	**IGF-1**		**IGF-2**	
**Biochemical parameters**	**Spearman correlation coefficient r**	**P value**	**Spearman correlation coefficient r**	**P value**
Glucose T0	0.165	0.542	0.234	0.336
Glucose T60	0.125	0.670	-0.021	0.937
Glucose T120	0.346	0.247	0.481	0.059
Insulin T0	0.466	0.127	0.693	0.006
Insulin T60	0.451	0.106	-0.087	0.740
Insulin T120	0.407	0.168	0.296	0.266
Adiponectin T0	-0.015	0.957	-0.001	0.997
Adiponectin T60	0.207	0.478	0.031	0.907
Adiponectin T120	0.392	0.208	-0.173	0.537
Apelin T0	0.315	0.253	0.067	0.793
Apelin T60	0.392	0.166	0.082	0.754
Apelin T120	0.363	0.203	0.145	0.580
Cholecystokinin T0	0.585	0.017	-0.033	0.892
Cholecystokinin T60	0.425	0.130	0.243	0.347
Cholecystokinin T120	0.405	0.151	0.223	0.389
Fibroblast growth factor 21 T0	0.112	0.703	0.296	0.266
Fibroblast growth factor 21 T60	0.203	0.505	0.071	0.800
Fibroblast growth factor 21 T120	0.291	0.334	0.075	0.790
Ghrelin T0	0.053	0.846	-0.501	0.029
Ghrelin T60	0.007	0.982	-0.481	0.051
Ghrelin T120	-0.026	0.929	-0.043	0.870
Glucagon-like peptide-1 T0	0.267	0.318	0.007	0.976
Glucagon-like peptide-1 T60	0.297	0.303	0.151	0.563
Glucagon-like peptide-1 T120	0.147	0.615	0.308	0.229
Leptin T0	0.379	0.147	0.356	0.135
Leptin T60	0.420	0.175	0.154	0.584
Leptin T120	0.455	0.138	-0.120	0.671
Leptin receptor T0	-0.156	0.564	-0.212	0.383
Leptin receptor T60	-0.222	0.446	-0.341	0.181
Leptin receptor T120	-0.279	0.334	-0.309	0.227
Resistin T0	-0.337	0.201	-0.053	0.829
Resistin T60	-0.121	0.681	-0.203	0.434
Resistin T120	0.055	0.858	-0.231	0.389
Visfatin T0	-0.051	0.864	-0.034	0.901
Visfatin T60	0.467	0.108	0.077	0.785
Visfatin T120	0.315	0.319	-0.040	0.893

Table S6. Correlations of biochemical parameters with IGF-1 and IGF-2 concentrations in the post-HSCT group.

	IGF-1		IGF-2	
**Biochemical parameters**	**Spearman correlation coefficient r**	**P value**	**Spearman correlation coefficient r**	**P value**
Glucose T0	-0.049	0.846	-0.049	0.842
Glucose T60	0.357	0.175	0.164	0.528
Glucose T120	0.458	0.086	0.097	0.72
Insulin T0	0.475	0.086	-0.115	0.694
Insulin T60	0.32	0.226	-0.101	0.699
Insulin T120	0.727	0.002	-0.153	0.57
Adiponectin T0	-0.09	0.722	-0.089	0.716
Adiponectin T60	-0.219	0.415	-0.063	0.81
Adiponectin T120	-0.518	0.06	-0.28	0.31
Apelin T0	0.186	0.475	0.38	0.12
Apelin T60	0.168	0.533	0.133	0.6
Apelin T120	0.182	0.5	0.16	0.54
Cholecystokinin T0	0.29	0.244	0.255	0.29
Cholecystokinin T60	0.325	0.22	0.18	0.479
Cholecystokinin T120	0.379	0.15	0.293	0.255
Fibroblast growth factor 21 T0	0.293	0.289	-0.064	0.8
Fibroblast growth factor 21 T60	0.303	0.293	-0.044	0.877
Fibroblast growth factor 21 T120	0.196	0.5	-0.058	0.84
Ghrelin T0	-0.26	0.292	0.013	0.96
Ghrelin T60	-0.154	0.569	0.159	0.542
Ghrelin T120	-0.194	0.473	0.081	0.756
Glucagon-like peptide-1 T0	0.223	0.375	0.158	0.519
Glucagon-like peptide-1 T60	0.313	0.238	0.114	0.664
Glucagon-like peptide-1 T120	0.338	0.2	0.206	0.427
Leptin T0	-0.084	0.74	-0.417	0.076
Leptin T60	-0.16	0.58	-0.645	0.009
Leptin T120	-0.178	0.543	-0.62	0.014
Leptin receptor T0	-0.324	0.189	0.042	0.867
Leptin receptor T60	-0.403	0.121	-0.016	0.95
Leptin receptor T120	-0.398	0.127	0.024	0.926
Resistin T0	0.274	0.27	0.316	0.188
Resistin T60	0.232	0.388	0.175	0.5
Resistin T120	0.106	0.708	0.19	0.48
Visfatin T0	0.33	0.228	0.014	0.96
Visfatin T60	0.5	0.065	0.104	0.713
Visfatin T120	0.37	0.21	-0.067	0.82

Table S7. Correlations of anthropometric parameters with IGFBP concentrations (pooled analysis).

	IGFBP-1		IGFBP-2		IGFBP-3		IGFBP-4		IGFBP-6		IGFBP-7	
**Parameter**	**Spearman correlation coefficient r**	**P value**	**Spearman correlation coefficient r**	**P value**	**Spearman correlation coefficient r**	**P value**	**Spearman correlation coefficient r**	**P value**	**Spearman correlation coefficient r**	**P value**	**Spearman correlation coefficient r**	**P value**
BMI percentile	-0.145	0.546	-0.247	0.442	-0.037	0.864	0.146	0.600	0.138	0.667	0.222	0.996
Blood pressure systolic/diastolic	-0.448/-0.494	0.024	-0.239/-0.257	0.442	-0.168/-0.22	0.571	-0.2/-0.203	0.472	0.234/0.409	0.492	-0.057/-0.036	0.996
Height mm	-0.626	0.0005	-0.157	0.598	0.211	0.454	-0.199	0.472	0.421	0.141	0.208	0.996
Weight kg	-0.6	0.0005	-0.174	0.576	0.144	0.648	-0.163	0.551	0.388	0.141	0.333	0.482
Waist circumference cm/percentile	-0.617/0.048	0.0005	-0.223/-0.044	0.442	0.185/0.11	0.544	-0.054/0.343	0.935	0.435/0.108	0.141	0.342/0.271	0.482
Age months	-0.586	0.001	-0.109	0.730	0.14	0.648	-0.233	0.472	-0.407	0.141	0.17	0.996
BMI WHO	-0.467	0.016	-0.265	0.442	0.042	0.864	0.066	0.907	0.318	0.222	0.42	0.423
BF kg/%	-0.558/-0.564	0.016	-0.185/-0.15	0.598	0.204/0.144	0.571	-0.186/-0.123	0.587	0.164/0.002	0.667	0.458/0.355	0.447
ECW	-0.455	0.059	-0.129	0.730	0.364	0.405	-0.293	0.472	0.222	0.654	0.295	0.967
LBM kg	-0.459	0.059	-0.165	0.643	0.292	0.405	-0.35	0.472	0.421	0.150	0.36	0.548
TBW	-0.464	0.059	-0.174	0.624	0.287	0.405	-0.352	0.472	0.424	0.150	0.376	0.548

Table S8. Correlations of anthropometric parameters with IGFBP concentrations in the pre-HSCT group.

	IGFBP-1		IGFBP-2		IGFBP-3		IGFBP-4		IGFBP-6		IGFBP-7	
**Parameter**	**Spearman correlation coefficient r**	**P value**	**Spearman correlation coefficient r**	**P value**	**Spearman correlation coefficient r**	**P value**	**Spearman correlation coefficient r**	**P value**	**Spearman correlation coefficient r**	**P value**	**Spearman correlation coefficient r**	**P value**
BMI percentile	-0.183	0.452	-0.547	0.023	0.330	0.167	-0.127	0.604	-0.152	0.535	-0.117	0.634
Blood pressure systolic/diastolic	-0.082/-0.270	0.740/0.262	-0.175/-0.173	0.501/0.506	0.001/0.040	0.997/0.869	-0.154/-0.036	0.530/0.883	-0.003/0.059	0.991/0.811	-0.036/0.111	0.884/0.652
Height mm	-0.484	0.036	-0.186	0.474	0.309	0.197	-0.427	0.068	0.812	0.000	0.532	0.019
Weight kg	-0.484	0.036	-0.284	0.269	0.330	0.168	-0.382	0.106	0.730	0.000	0.607	0.006
Waist circumference cm/percentile	-0.518/-0.040	0.023/0.887	-0.350/-0.448	0.168/0.125	0.403/0.190	0.087/0.498	-0.365/0.237	0.124/0.395	0.619/-0.171	0.005/0.541	0.491/-0.226	0.033/0.418
Age months	-0.401	0.089	-0.114	0.663	0.233	0.336	-0.430	0.066	0.780	0.000	0.556	0.014
BMI WHO	-0.415	0.077	-0.339	0.184	0.302	0.209	-0.310	0.197	0.335	0.162	0.270	0.263
BF kg/%	-0.552/-0.663	0.050/0.014	-0.375/-0.529	0.230/0.077	0.263/0.055	0.385/0.857	-0.309/-0.188	0.304/0.539	0.536/0.342	0.059/0.252	0.326/0.271	0.277/0.371
ECW	-0.418	0.156	-0.203	0.527	0.479	0.097	-0.522	0.067	0.687	0.010	0.687	0.010
LBM kg	-0.132	0.668	-0.175	0.587	0.562	0.046	-0.484	0.094	0.736	0.004	0.637	0.019
TBW	-0.132	0.668	-0.175	0.587	0.562	0.046	-0.484	0.094	0.736	0.004	0.637	0.019

Table S9. Correlations of anthropometric parameters with IGFBP concentrations in the post-HSCT group.

	IGFBP-1		IGFBP-2		IGFBP-3		IGFBP-4		IGFBP-6		IGFBP-7	
**Parameter**	**Spearman correlation coefficient r**	**P value**	**Spearman correlation coefficient r**	**P value**	**Spearman correlation coefficient r**	**P value**	**Spearman correlation coefficient r**	**P value**	**Spearman correlation coefficient r**	**P value**	**Spearman correlation coefficient r**	**P value**
BMI percentile	-0.086	0.727	-0.222	0.392	-0,338	0,157	-0,102	0,679	0,518	0,023	-0,044	0,858
Blood pressure systolic/diastolic	-0.62/-0.634	0.005/0.003	-0.265/-0.302	0.3/0.24	-0,08/-0.477	0,745/0.039	-0,37/-0.443	0,119/0.058	0,504/0.812	0,028/0.000	-0,03/-0.29	0,903/0.411
Height mm	-0.79	0.001	-0.258	0.317	0,074	0,763	-0,077	0,753	0,203	0,405	0,067	0,786
Weight kg	-0.754	0.002	-0.268	0.299	-0,017	0,944	-0,069	0,778	0,301	0,210	0,083	0,734
Waist circumference cm/percentile	-0.71/-0.486	0.001/0.056	-0.229/-0.095	0.376/0.758	-0,076/0.045	0,759/0.87	-0,014/0.257	0,955/0.355	0,532/0.384	0,019/0.158	0,049/0.29	0,842/0.294
Age months	-0.747	0.000	-0.238	0.358	-0,043	0,861	-0,075	0,759	0,238	0,327	0,003	0,991
BMI WHO	-0.525	0.02	-0.317	0.215	-0,281	0,244	-0,076	0,756	0,591	0,008	-0,08	0,745
BF kg/%	-0.634/-0.425	0.02/0.148	-0.247/-0.079	0.44/0.807	0,34/0.127	0,255/0.679	0,099/0.168	0,747/0.584	-0,088/-0.05	0,775/0.865	0,342/0.369	0,253/0.215
ECW	-0.514	0.072	-0.217	0.498	0,455	0,118	0,132	0,667	0,005	0,986	0,335	0,263
LBM kg	-0.668	0.013	-0.277	0.383	0,257	0,397	-0,126	0,68	0,335	0,263	0,055	0,859
TBW	-0.665	0.013	-0.282	0.373	0,242	0,426	-0,129	0,674	0,341	0,254	0,052	0,865

Table S10. Correlations of biochemical parameters with IGFBP concentrations (pooled analysis)

	IGFBP-1		IGFBP-2		IGFBP-3		IGFBP-4		IGFBP-6		IGFBP-7	
**Parameter**	**Spearman correlation coefficient r**	**P value**	**Spearman correlation coefficient r**	**P value**	**Spearman correlation coefficient r**	**P value**	**Spearman correlation coefficient r**	**P value**	**Spearman correlation coefficient r**	**P value**	**Spearman correlation coefficient r**	**P value**
Glucose T0	-0.189	0.462	-0.288	0.406	-0.117	0.715	-0.189	0.481	0.18	0.654	0.057	0.996
Glucose T60	-0.02	0.940	-0.032	0.924	0.121	0.715	-0.244	0.472	0.137	0.667	-0.198	0.996
Glucose T120	-0.2	0.462	-0.173	0.598	0.298	0.405	0.023	0.980	0.281	0.426	0.156	0.996
Insulin T0	-0.457	0.059	-0.421	0.213	0.01	0.960	0.12	0.783	0.067	0.819	-0.003	0.996
Insulin T60	-0.397	0.059	-0.293	0.406	0.111	0.716	-0.174	0.551	0.062	0.819	-0.12	0.996
Insulin T120	-0.429	0.059	-0.297	0.406	0.235	0.454	-0.007	0.980	0.142	0.667	-0.077	0.996
Adiponectin T0	0.073	0.779	-0.093	0.747	-0.092	0.735	-0.106	0.783	0.145	0.667	0.097	0.996
Adiponectin T60	-0.038	0.887	-0.246	0.442	-0.25	0.405	-0.18	0.551	-0.079	0.819	0.105	0.996
Adiponectin T120	-0.102	0.732	-0.173	0.598	-0.302	0.405	-0.045	0.935	0.078	0.819	-0.048	0.996
Apelin T0	-0.015	0.940	0.108	0.730	0.264	0.405	-0.228	0.472	0.179	0.654	-0.074	0.996
Apelin T60	-0.048	0.863	-0.05	0.879	0.385	0.353	-0.219	0.472	0.033	0.892	0.186	0.996
Apelin T120	-0.05	0.863	0.061	0.845	0.37	0.353	-0.235	0.472	0.062	0.819	0.006	0.996
Cholecystokinin T0	-0.133	0.560	-0.02	0.953	0.3	0.405	-0.233	0.472	0.225	0.511	-0.011	0.996
Cholecystokinin T60	-0.14	0.560	0.062	0.845	0.344	0.405	-0.232	0.472	0.19	0.654	-0.076	0.996
Cholecystokinin T120	-0.15	0.546	0.003	0.999	0.394	0.353	-0.261	0.472	0.174	0.664	-0.077	0.996
Fibroblast growth factor 21 T0	-0.32	0.165	-0.222	0.478	-0.203	0.544	-0.258	0.472	0.206	0.654	-0.088	0.996
Fibroblast growth factor 21 T60	-0.365	0.128	-0.252	0.442	-0.252	0.443	-0.257	0.472	0.115	0.753	-0.103	0.996
Fibroblast growth factor 21 T120	-0.338	0.165	-0.247	0.442	-0.287	0.405	-0.221	0.472	0.137	0.690	-0.105	0.996
Ghrelin T0	0.321	0.128	0.29	0.406	0.111	0.715	-0.049	0.935	0.024	0.903	-0.05	0.996
Ghrelin T60	0.289	0.200	0.506	0.080	0.182	0.571	-0.1	0.783	0.044	0.860	-0.093	0.996
Ghrelin T120	0.295	0.200	0.369	0.213	0.173	0.571	-0.106	0.783	-0.093	0.782	-0.12	0.996
Glucagon-like peptide-1 T0	-0.16	0.546	-0.103	0.730	0.261	0.405	-0.092	0.783	-0.353	0.150	-0.01	0.996
Glucagon-like peptide-1 T60	-0.16	0.546	-0.03	0.924	0.42	0.353	-0.212	0.472	0.319	0.259	0.05	0.996
Glucagon-like peptide-1 T120	-0.23	0.357	-0.19	0.552	0.275	0.405	-0.257	0.472	0.267	0.426	-0.004	0.996
Leptin T0	-0.257	0.233	-0.237	0.442	0.134	0.661	0.247	0.472	0.134	0.667	-0.014	0.996
Leptin T60	-0.224	0.423	-0.421	0.208	0.101	0.735	0.282	0.472	0.157	0.667	-0.008	0.996
Leptin T120	-0.165	0.546	-0.392	0.213	0.083	0.780	0.357	0.472	0.177	0.667	-0.035	0.996
Leptin receptor T0	0.6	0.001	0.184	0.552	0.03	0.878	-0.048	0.935	-0.354	0.150	-0.148	0.996
Leptin receptor T60	0.774	<0.001	0.246	0.442	0.043	0.864	-0.036	0.939	-0.404	0.141	-0.17	0.996

Leptin receptor T120	0.712	<0.001	0.227	0.442	0.039	0.864	0.042	0.935	-0.406	0.141	-0.173	0.996
Resistin T0	0.275	0.200	0.412	0.154	0.058	0.864	-0.004	0.980	-0.019	0.910	-0.012	0.996
Resistin T60	0.05	0.863	0.454	0.108	0.09	0.736	0.008	0.980	-0.057	0.819	-0.032	0.996
Resistin T120	0.014	0.940	0.499	0.080	0.119	0.715	0.015	0.980	-0.114	0.753	-0.111	0.996
Visfatin T0	-0.083	0.779	0.08	0.808	0.269	0.405	-0.229	0.472	0.187	0.654	0.001	0.996
Visfatin T60	-0.167	0.546	0.0002	0.999	0.289	0.405	-0.286	0.472	0.074	0.819	0.199	0.996
Visfatin T120	-0.124	0.673	0.106	0.747	0.118	0.718	-0.282	0.472	0.111	0.772	0.034	0.996

Table S11. Correlations of biochemical parameters with IGFBP concentrations in the pre-HSCT group

	**IGFBP-1**		**IGFBP-2**		**IGFBP-3**		**IGFBP-4**		**IGFBP-6**		**IGFBP-7**	
**Parameter**	**Spearman correlation coefficient r**	**P value**	**Spearman correlation coefficient r**	**P value**	**Spearman correlation coefficient r**	**P value**	**Spearman correlation coefficient r**	**P value**	**Spearman correlation coefficient r**	**P value**	**Spearman correlation coefficient r**	**P value**
Glucose T0	-0.232	0.340	-0.194	0.456	-0.121	0.621	-0.058	0.814	0.349	0.143	0.016	0.949
Glucose T60	-0.097	0.721	-0.475	0.074	0.473	0.064	-0.153	0.572	0.329	0.213	0.338	0.200
Glucose T120	0.461	0.063	0.241	0.368	0.431	0.084	-0.311	0.224	0.174	0.504	-0.091	0.729
Insulin T0	-0.275	0.341	-0.543	0.068	0.358	0.208	-0.097	0.742	0.447	0.109	-0.057	0.846
Insulin T60	-0.191	0.462	-0.288	0.279	0.675	0.003	-0.578	0.015	0.360	0.155	-0.115	0.660
Insulin T120	-0.338	0.200	-0.489	0.064	0.473	0.064	-0.094	0.729	0.403	0.122	-0.241	0.368
Adiponectin T0	-0.088	0.721	-0.319	0.213	-0.011	0.963	-0.225	0.355	0.333	0.163	0.265	0.273
Adiponectin T60	-0.229	0.376	-0.477	0.062	-0.145	0.579	-0.153	0.557	0.246	0.340	0.233	0.368
Adiponectin T120	-0.043	0.864	-0.789	0.000	0.390	0.110	-0.221	0.378	0.076	0.763	0.090	0.723
Apelin T0	-0.268	0.334	-0.279	0.334	-0.106	0.708	0.207	0.459	0.243	0.383	-0.039	0.889
Apelin T60	-0.069	0.793	-0.730	0.001	0.414	0.098	-0.239	0.355	-0.052	0.844	0.060	0.819
Apelin T120	-0.049	0.852	-0.606	0.013	0.427	0.087	-0.191	0.462	-0.132	0.613	0.049	0.852
Cholecystokinin T0	-0.208	0.394	-0.515	0.034	0.193	0.429	-0.062	0.802	0.264	0.275	0.090	0.715
Cholecystokinin T60	0.082	0.754	-0.521	0.039	0.133	0.610	0.053	0.841	0.349	0.169	-0.097	0.711
Cholecystokinin T120	-0.247	0.356	-0.443	0.098	-0.223	0.407	-0.121	0.656	0.032	0.905	0.262	0.327
Fibroblast growth factor 21 T0	-0.271	0.293	-0.518	0.040	0.309	0.228	-0.349	0.169	0.349	0.169	0.175	0.501
Fibroblast growth factor 21 T60	-0.386	0.156	-0.402	0.154	-0.277	0.317	-0.229	0.413	0.014	0.960	0.129	0.648
Fibroblast growth factor 21 T120	-0.382	0.160	-0.354	0.215	-0.304	0.270	-0.296	0.283	-0.029	0.919	0.121	0.666
Ghrelin T0	0.133	0.586	0.547	0.023	-0.278	0.250	0.130	0.596	-0.091	0.710	0.132	0.591
Ghrelin T60	0.159	0.541	0.509	0.044	-0.227	0.381	0.098	0.708	-0.032	0.903	0.181	0.486
Ghrelin T120	0.464	0.061	0.648	0.007	-0.160	0.539	0.078	0.765	-0.039	0.881	-0.197	0.448
Glucagon-like peptide-1 T0	0.016	0.949	-0.529	0.029	0.199	0.414	-0.361	0.129	0.140	0.566	0.272	0.260
Glucagon-like peptide-1 T60	-0.119	0.649	-0.623	0.010	0.356	0.160	-0.352	0.166	0.104	0.691	0.113	0.666
Glucagon-like peptide-1 T120	-0.181	0.486	-0.532	0.034	0.179	0.491	-0.414	0.098	0.174	0.504	0.157	0.548
Leptin T0	-0.332	0.166	-0.532	0.028	0.404	0.086	-0.196	0.420	0.523	0.022	0.225	0.355
Leptin T60	-0.443	0.098	-0.771	0.001	0.422	0.117	-0.304	0.271	0.382	0.160	0.161	0.567
Leptin T120	-0.325	0.237	-0.591	0.026	0.466	0.080	-0.368	0.177	0.271	0.328	0.246	0.376
Leptin receptor T0	0.363	0.126	0.002	0.993	-0.452	0.052	0.091	0.710	-0.649	0.003	-0.326	0.173

Leptin receptor T60	0.346	0.174	0.097	0.721	-0.603	0.010	0.059	0.823	-0.718	0.001	-0.174	0.504
Leptin receptor T120	0.353	0.165	0.082	0.762	-0.608	0.010	0.157	0.548	-0.738	0.001	-0.338	0.184
Resistin T0	0.587	0.008	0.629	0.007	0.096	0.695	0.089	0.718	0.075	0.761	0.128	0.601
Resistin T60	0.466	0.059	0.765	0.001	0.029	0.912	0.023	0.929	0.013	0.959	0.060	0.819
Resistin T120	0.492	0.053	0.777	0.001	0.094	0.728	0.004	0.987	-0.004	0.987	-0.078	0.774
Visfatin T0	0.050	0.854	-0.615	0.015	0.148	0.584	0.215	0.424	-0.004	0.987	0.149	0.583
Visfatin T60	-0.118	0.676	-0.714	0.004	0.097	0.732	0.164	0.558	0.161	0.567	0.139	0.621
Visfatin T120	-0.275	0.342	-0.670	0.012	-0.352	0.217	0.314	0.274	-0.218	0.455	0.393	0.164

Table S12. Correlations of biochemical parameters with IGFBP concentrations in the post-HSCT group

	IGFBP-1		IGFBP-2		IGFBP-3		IGFBP-4		IGFBP-6		IGFBP-7	
**Parameter**	**Spearman correlation coefficient r**	**P value**	**Spearman correlation coefficient r**	**P value**	**Spearman correlation coefficient r**	**P value**	**Spearman correlation coefficient r**	**P value**	**Spearman correlation coefficient r**	**P value**	**Spearman correlation coefficient r**	**P value**
Glucose T0	-0.03	0.9	-0.47	0.057	-0.13	0.596	-0.382	0.106	0,091	0,710	-0,009	0,972
Glucose T60	-0.267	0.3	-0.085	0.756	-0.433	0.083	-0.35	0.168	0,216	0,406	-0,267	0,3
Glucose T120	-0.28	0.288	-0.2	0.469	-0.26	0.332	-0.139	0.6	0,104	0,700	-0,238	0,374
Insulin T0	-0.7	0.005	-0.2	0.53	-0.213	0.465	-0.097	0.84	0,048	0,869	-0,233	0,422
Insulin T60	-0.444	0.07	-0.235	0.38	-0.403	0.108	0.022	0.933	0,294	0,251	-0,321	0,208
Insulin T120	-0.497	0.05	-0.16	0.56	-0.245	0.362	0.012	0.966	0,144	0,594	-0,062	0,820
Adiponectin T0	0.114	0.64	0.359	0.157	-0.227	0.351	0.054	0.355	0,112	0,647	-0,128	0,601
Adiponectin T60	0.31	0.227	0.15	0.57	-0.472	0.056	0.44	0.866	-0,137	0,599	0,075	0,775
Adiponectin T120	0.35	0.21	0.217	0.456	-0.384	0.157	0.246	0.376	0,139	0,621	0,057	0,84
Apelin T0	-0.05	0.84	0.1	0.71	0.13	0.6	-0.47	0.048	0,069	0,785	-0,104	0,681
Apelin T60	-0.194	0.455	-0.008	0.978	0.348	0.172	-0.358	0.158	-0,069	0,793	0,26	0,314
Apelin T120	-0.218	0.4	-0.059	0.823	0.357	0.159	-0.43	0.084	-0,002	0,993	0,181	0,486
Cholecystokinin T0	-0.26	0.276	-0.12	0.64	0,205	0,399	-0.643	0.003	0,373	0,116	-0,156	0,523
Cholecystokinin T60	-0.338	0.18	-0.045	0.867	0,424	0,09	0.627	0.007	0,13	0,619	-0,103	0,694
Cholecystokinin T120	-0.325	0.2	-0.122	0.65	0,517	0,033	-0.571	0.017	0,11	0,673	0,017	0,948
Fibroblast growth factor 21 T0	-0.273	0.31	0.118	0.677	-0,025	0,927	-0.247	0.356	0,197	0,464	0,156	0,564
Fibroblast growth factor 21 T60	-0.32	0.245	0.04	0.89	-0,068	0,81	-0.103	0.713	0,254	0,362	0,068	0,810
Fibroblast growth factor 21 T120	-0.223	0.425	0.01	0.97	-0,397	0,143	-0.318	0.248	0,45	0,092	-0,043	0,879
Ghrelin T0	0.435	0.063	0.189	0.467	0,036	0,884	-0.019	0.937	-0,019	0,937	-0,018	0,943
Ghrelin T60	0.321	0.21	0.413	0.112	0,09	0,733	0.08	0.75	-0,061	0,815	0,14	0,593
Ghrelin T120	0.352	0.166	0.27	0.313	0,205	0,43	0.027	0.918	-0,240	0,353	0,186	0,474
Glucagon-like peptide-1 T0	-0.33	0.167	-0.215	0.409	-0,11	0,655	-0,465	0,045	0,553	0,014	-0,135	0,581

Glucagon-like peptide-1 T60	-0.45	0.07	-0.163	0.547	0,189	0,468	-0,495	0,043	0,311	0,224	0,098	0,708
Glucagon-like peptide-1 T120	-0.423	0.091	-0.328	0.22	-0,046	0,86	-0,64	0,006	0,451	0,069	0,009	0,974
Leptin T0	-0.285	0.237	-0.237	0.36	-0,479	0,038	-0,177	0,468	0,491	0,033	-0,16	0,514
Leptin T60	-0.159	0.57	-0.33	0.246	-0,259	0,351	-0,064	0,82	0,536	0,04	0,018	0,95
Leptin T120	-0.1	0.7	-0.372	0.19	-0,511	0,051	0,068	0,81	0,593	0,02	-0,182	0,516
Leptin receptor T0	0.6	0.006	0.256	0.32	0,236	0,332	-0,068	0,781	-0,266	0,271	0,018	0,943
Leptin receptor T60	0.756	0.0004	0.097	0.721	0,266	0,302	-0,005	0,985	-0,539	0,026	0,064	0,808
Leptin receptor T120	0.657	0.004	0.317	0.232	0,353	0,164	-0,015	0,955	-0,542	0,025	0,017	0,948
Resistin T0	-0.123	0.6	0.309	0.227	0,076	0,756	0,251	0,3	-0,054	0,825	0,012	0,960
Resistin T60	-0.184	0.48	0.2	0.45	0,175	0,51	0,314	0,22	-0,213	0,411	0,255	0,323
Resistin T120	-0.197	0.464	0.304	0.271	0,29	0,276	0,372	0,156	-0,256	0,338	0,246	0,359
Visfatin T0	-0.3	0.257	0.07	0.8	-0,04	0,883	-0,753	0,001	0,147	0,587	-0,097	0,721
Visfatin T60	-0.413	0.126	0.02	0.946	0,086	0,761	-0,711	0,003	-0,121	0,666	0,139	0,621
Visfatin T120	-0.34	0.235	0.077	0.802	-0,09	0,759	-0,789	0,001	0,037	0,899	-0,108	0,714
